# The Characteristics and Significance of Locally Infiltrating B Cells in Lupus Nephritis and Their Association with Local BAFF Expression

**DOI:** 10.1155/2013/954292

**Published:** 2013-06-12

**Authors:** Chuan-Yin Sun, Yan Shen, Xiao-Wei Chen, Yu-Cheng Yan, Feng-Xia Wu, Ming Dai, Ting Li, Cheng-De Yang

**Affiliations:** ^1^Department of Rheumatology, Renji Hospital, Shanghai Jiaotong University School of Medicine, 145 Shan Dong Zhong Road, Shanghai 200001, China; ^2^Department of Rheumatology, First Affiliated Hospital of Medical School, Zhejiang University, 79 Qingchun Road, Hangzhou 310003, China; ^3^Department of Nephrology, Renji Hospital, Shanghai Jiaotong University School of Medicine, 145 Shan Dong Zhong Road, Shanghai 200001, China

## Abstract

*Introduction.* Dysfunction of the B lymphocyte is considered to be involved in the pathogenesis of lupus nephritis (LN). Intrarenal B cells have been found in several forms of inflammatory kidney disease. B-cell activating factor (BAFF) regulates B lymphocyte proliferation and survival, and contributes to human autoimmune disease. Their role in renal inflammation is not well defined. *Methods.* Clinical parameters and renal biopsies from 62 LN patients were prospectively analyzed. We performed standard immunohistochemistry on serial paraffin tissue sections using monoclonal antibodies to CD20 and BAFF to investigate the characteristics and significance of locally infiltrating B cells and local BAFF expression in patients with LN. *Results.* Intrarenal B cells and/or BAFF were mainly distributed in the renal interstitium. Compared to the LN-non-B-cell/BAFF expression group, proteinuria (g/24 hour), blood urea nitrogen, serum creatinine levels, LN renal activity, and chronicity indices, were all significantly greater in the LN-B-cell/BAFF expression groups. The expression of BAFF was strongly associated with the quantity of B-cell infiltrate in the interstitium. *Conclusion.* As BAFF expression was strongly associated with B-cell infiltration, we hypothesize that altered B-cell differentiation and tolerance induced by excess BAFF may be central to the pathogenesis of LN.

## 1. Introduction

Lupus nephritis (LN) in systemic lupus erythematosus (SLE) is a major cause of morbidity and end-stage renal disease [1]. LN develops in up to 60% of SLE patients during the course of the disease [2]. Dysfunction of B cells is thought to be important in the pathogenesis of SLE. B cells are also considered to be involved in LN, particularly as a source of nephritogenic auto-antibodies [3].

Intrarenal inflammation is a common feature in LN. However, little is known about the role of B cells as part of the infiltrating cell population. This might be because B cells have classically been considered to exert long-range effects, mostly via activation in secondary lymphoid organs such as lymph nodes and the spleen, with subsequent proliferation and differentiation into antibody-producing plasma cells. 

Studies have described the high prevalence of intrarenal B cells in immune-mediated diseases, including renal transplant rejection and glomerulonephritis [4–6]. Local B-cell infiltrates could play a role in tissue injury such as tissue fibrosis, neolymphangiogenesis, and ectopic lymphomagenesis [7]. Recently, a contribution of B cells to the formation of lymphoid-like structures in renal tissue has been proposed [8]. Steinmetz et al. [9] first examined B cells in LN patients and evaluated that most B cells displayed a mature non-antibody producing phenotype with antigen presenting ability. These findings led us to hypothesize on their functional importance. Intrarenal B cells may be part of a local system that plays a pivotal role in the pathogenesis of LN. 

B-cell activating factor (BAFF, also known as B-lymphocyte stimulator, BLyS) belongs to the tumor necrosis factor (TNF) superfamily and can be produced by myeloid cells such as monocytes, macrophages, dendritic cells, and neutrophils. BAFF contributes to B-cell proliferation and differentiation, and it is important in immunoglobulin class switching [10]. Many researchers have demonstrated that high levels of BAFF may relax B-cell selection and contribute to autoantibody production, exacerbating proteinuria and renal inflammation in SLE [11]. Tissue expression of BAFF has been found in germinal center B cells and/or plasma cells in lymph nodes of patients with SLE [12]. Neusser et al. [13] was the first to investigate BAFF in the kidneys of LN patients and found that BAFF was expressed in the interstitial inflammatory cell accumulation. We hypothesize that altered B-cell differentiation and tolerance induced by excess BAFF expression may be central to the pathogenesis of LN.

In this study, renal B-cell infiltrates and BAFF expression were analyzed in human LN patients. By doing so, the relationship between B-cell infiltration and BAFF expression could potentially elucidate the mechanisms underlying LN.

## 2. Patients and Methods

### 2.1. Patients

A prospective study of 62 patients who attended the Department of Rheumatology of Renji Hospital at the Shanghai Jiaotong University School of Medicine was undertaken. All patients fulfilled the American College of Rheumatology classification criteria for the diagnosis of SLE [14]. Clinical evidence of LN was obtained in all cases, and pathologic findings from renal biopsy specimens confirmed the diagnosis. Plasma samples were collected on the day of the renal biopsy. The following demographic, clinical, and serologic data were collected at the time of renal biopsy: sex; age; duration of SLE and LN; Systemic Lupus Erythematosus Disease Activity Index (SLEDAI); levels of proteinuria, blood urea nitrogen, and serum creatinine; and serum C3, C4, antinuclear antibodies (ANA), anti-Sm, antiribonucleoprotein (anti-RNP), antidouble-stranded DNA (anti-dsDNA), and anti-nucleosome antibodies.

The patients were informed of the purpose of the study and gave their consent for participation. The study was approved by the institutional review board of Shanghai Jiaotong University.

### 2.2. Enzyme-Linked Immunosorbent Assay (ELISA)

Plasma BAFF levels from 62 patients with LN and 40 healthy controls were detected using ELISA kits according to the manufacturer's instructions (F00153, Westang, China).

### 2.3. Biopsy Samples

#### 2.3.1. Histology of Renal Biopsy Samples

All patients underwent ultrasound-guided renal needle biopsy. Renal tissue obtained was fixed in 10% neutral buffered formalin, dehydrated gradually, and embedded in paraffin. Paraffin-embedded tissue sections were stained with hematoxylin and eosin, periodic acid-Schiff, Masson's trichrome, and periodic acid-silver methenamine. Small portions of fresh renal tissue were snap-frozen, and 4 *μ*m cryostat-cut sections were incubated with fluorescein isothiocyanate- (FITC-) conjugated rabbit antisera against human IgG, IgA, IgM, C1q, and C3 (Dako, Denmark), and direct immunofluorescence of these sections was examined. The biopsy specimens were classified using the International Society of Nephrology/Renal Pathology Society (ISN/RPS) 2003 classification of LN [15].

#### 2.3.2. Activity and Chronicity Indices of Renal Tissue Injury

Renal tissue injury was evaluated on the basis of activity and chronicity indices according to methods reported by Austin et al. [16]. The activity index was calculated as the sum of the scores (on a scale of 1–3) of endocapillary proliferation, karyorrhexis, fibrinoid necrosis (the score was multiplied by 2), cellular crescents (the score was multiplied by 2), hyaline deposits, leukocyte exudation, and interstitial inflammation. The chronicity index was the sum of the scores (on a scale of 1–3) for glomerular sclerosis, fibrous crescents, tubular atrophy, and interstitial fibrosis.

#### 2.3.3. Immunohistochemical Staining of Renal Biopsy Samples

As previously described [9], we used serial sections for each patient. CD20 monoclonal antibody (L26, Dako; Glostrup, Denmark) and BAFF monoclonal antibody (ALX-804-131-C100, Enzo Life Sciences International Inc.) were used as immunohistochemical staining to identify infiltrating B cells and BAFF expression. Immunohistochemistry was performed as previously described [9]. Briefly, the paraffin-embedded tissue sections were placed on positively charged slides and incubated in a stove at 60°C for one hour. The sections were deparaffinized and rehydrated through a series of washes with xylene and graded alcohol. Endogenous peroxidase was blocked by treatment with 3% H_2_O_2_ for 30 minutes. Antigen retrieval was performed by flooding the slides with EDTA-Tris buffer (pH 9.6) and heating in a microwave at 1,000 W for 10 minutes. Primary rat monoclonal anti-human BAFF antibody was applied to the slides at a dilution of 1 : 50 in 1% bovine serum albumin/phosphate buffered saline (BSA/PBS), and the slides were subsequently incubated overnight at room temperature. The slides were then incubated with a secondary goat anti-rat IgM antibody (ALX-211-055-R050, Enzo Life Sciences International Inc.) for 30 minutes. These sections were then washed with PBS (pH 7.4) between each step (3 times, 5 minutes each time). Finally, the sections were counterstained with Mayer's hematoxylin, air-dried, cleared in xylene, and coverslipped. Positive controls were performed on sections of healthy human lymph node with a nonimmune rabbit serum. The slides were counterstained with haematoxylin and observed under a Leica light microscope. In light microscopy positive parts show brown.

#### 2.3.4. Quantification of Immunofluorescence and Immunohistochemical Staining Scores

**Figure 1:**
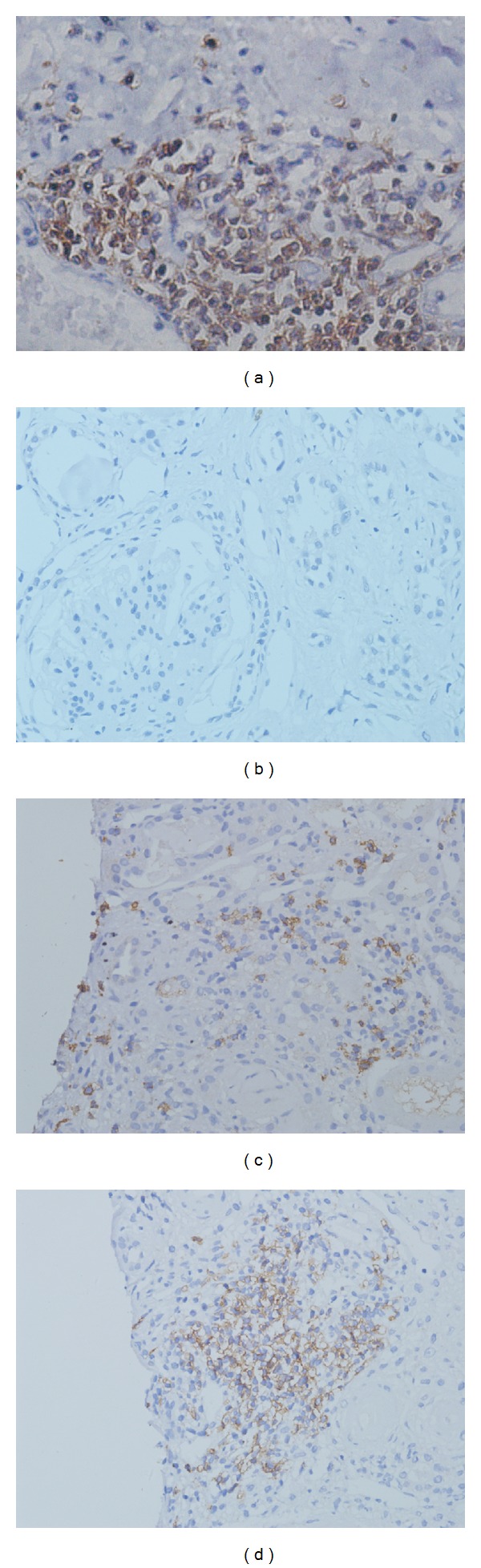
Microanatomical organization of B cells infiltrates. B-cells show a cluster-like structure in the lymph node of a healthy control (a). Serial staining for CD20 allowed classification into three different grades: grade 0, no infiltrate of intrarenal CD20-positive B cells (b); grade 1, a scattered pattern of intrarenal CD20-positive B cells (c); and grade 2, nodular aggregates pattern of CD20-positive B cells (d). (Original magnification: ×400).

**Figure 2:**
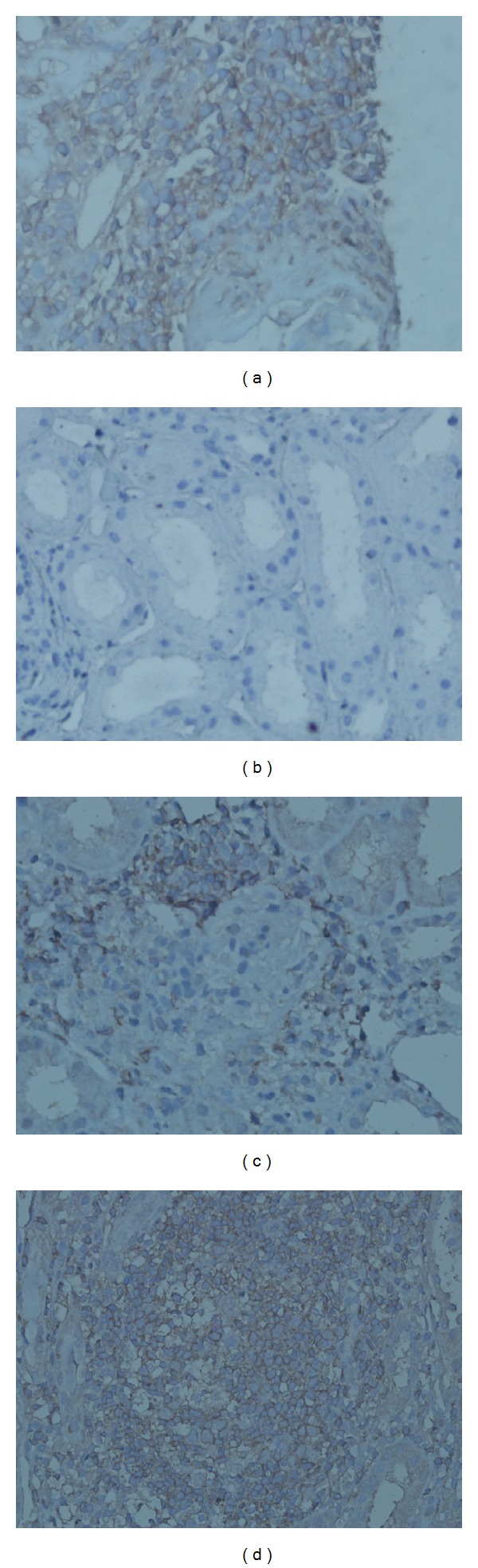
Enhanced BAFF expression in tissue samples. High levels of BAFF expression in the lymph node of a healthy control (a). BAFF expression in renal biopsies with lupus nephritis: no staining (b), mild staining (c), and moderate to intense staining (d). (Original magnification: ×400).

Results of the renal biopsies from the 62 patients with LN were classified according to the organizational stage of B-cell infiltrates. Three distinct categories could be distinguished: no infiltrate of intrarenal CD20-positive B cells was graded as 0 (group 0); a scattered pattern of intrarenal CD20-positive B cells was graded as 1 (group 1); and nodular aggregates consisting of CD20-positive B cells were graded as 2 (group 2). Typical examples of different grades infiltrates are shown in Figures 1(a)–1(d). The results of intrarenal staining for BAFF were scored as 0, 1, or 2, where 0 represented absence of BAFF staining, 1 represented weak BAFF staining, and 2 represented moderate or strong staining. Typical examples of BAFF expression in consecutive tissue samples are shown in Figures 2(a)–2(d). All the aforementioned biopsy specimens were scored by a renal pathologist who had no prior knowledge of the clinical and laboratory findings of the patients. 

### 2.4. Statistical Analysis

SPSS version 11.0 software (SPSS Inc., USA) was used to perform all statistical analyses. Categorical variables were compared using Fisher's exact test or the chi-square test. Differences between the median values of defined patient groups were compared using the nonparametric Mann-Whitney *U* test. A Spearman's rank correlation was used to detect correlations between different study parameters. A *P* value < 0.05 was considered statistically significant.

## 3. Results

### 3.1. Demographic, Clinical Characteristics and Laboratory Results of the LN Patients

In this study, we prospectively examined 62 LN patients (58 women and 4 men) with a mean age ± SD of 31 ± 11 years. Depending on the presence or not of B-cell infiltrates in renal biopsies, the patients were divided into two groups, that is, those with B-cell infiltration (the LN-B group) and those without (LN-non-B group). There were 35 (56.5%) patients in the LN-B group (32 women and 3 men) and 27 (43.5%) patients in the LN-non-B group (26 women and 1 man). The mean age ± SD of the two groups was 33 ± 11 and 29 ± 11 years, respectively. No significant difference was seen between the groups in terms of age or gender (*P* > 0.05 for all). Proteinuria (g/24 hours), blood urea nitrogen, and serum creatinine levels were all significantly greater in the LN-B cell group than in the LN-non-B cell group (*P* < 0.05 for all). However, the duration of SLE or LN, SLEDAI, as well as the level of C3/C4, was not statistically different between the two groups. In addition, we failed to find any association between the presence of intrarenal B-cell infiltration and that of anti-dsDNA antibodies (*P* > 0.05) (Table 1).

### 3.2. Clinical Features of Different Staining Grades

**Figure 3:**
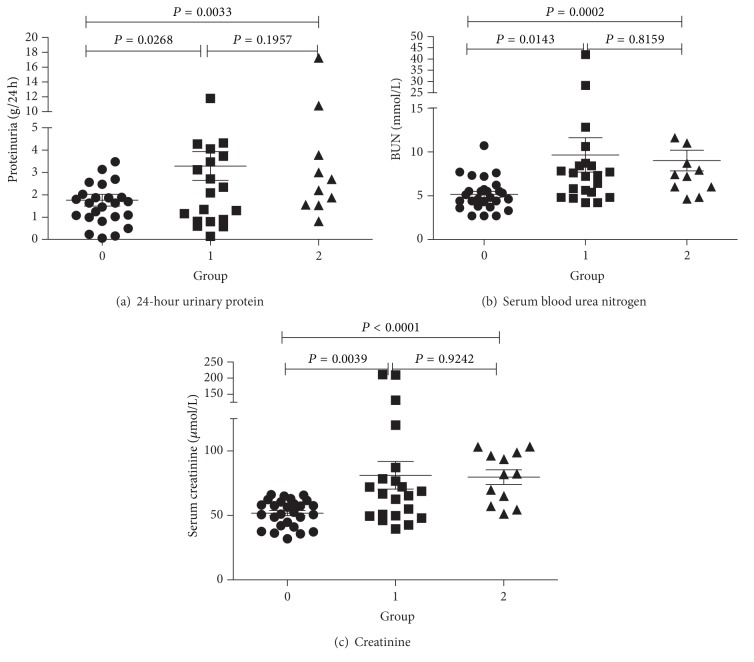
Clinical parameters in different groups. 24-hour urinary protein (a), serum blood urea nitrogen (b), and creatinine (c) levels in three different B-cell groups of LN patients. *P* < 0.05 was considered statistically significant.

We examined renal biopsy samples of all 62 patients. Using B-cell immunohistochemical staining, 27 (43.5%) patients were classified grade 0, 23 (37.1%) grade 1, and 12 (19.4%) grade 2. We found that the level of proteinuria (g/24 hours) in grade 0 was lower than other grades (*P* < 0.05 for all), and there was no statistically difference between grade 1 and 2 (*P* > 0.05 for all). The same results in serum creatinine and blood urea nitrogen level (Figure 3). 

### 3.3. Microanatomical Organization of Inflammatory Infiltrates

All biopsy samples from the patients were stained for CD20 as a pan B-cell marker. B cells were mainly distributed in the renal tubules, the renal interstitial vessels, and in the interstitium, but rarely in glomerular tissue (Figure 1). 

### 3.4. Inflammatory Infiltrates and Renal Histology

**Figure 4:**
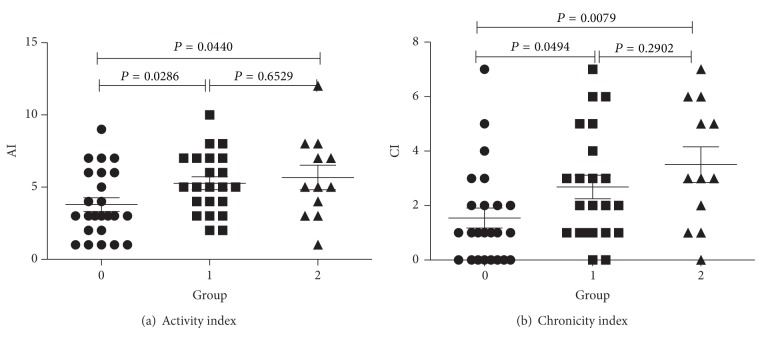
Clinical parameters in different groups. Lupus nephritis activity index (a) and chronicity index (b) in three different B-cell groups of LN patients. *P* < 0.05 was considered statistically significant.

The distribution of the ISN/RPS classification of the patients was as follows: 3 were class I, 2 were class II, 4 were class III, 22 were class IV, 14 were class V, 11 were class (III + V), and 6 were class (IV + V). In the LN-B-cell group, none of the biopsies were class I or class II, 3 were class III, 17 were class IV, 6 were class V, 4 were class III + V, and 5 were class IV + V. When compared with the LN-non-B-cell group, the LN-B-cell group was more likely to be associated with class IV LN (*P* = 0.0142) (Table 2). Within the three different groups of LN patients, we found that the activity and chronicity indices of grade 0 were significantly lower than those of the other grades (*P* < 0.05 for all), but there was no significant difference between grade 1 and grade 2 (*P* > 0.05 for all) (Figure 4). 

### 3.5. Characterization of Intrarenal BAFF Expression

For BAFF expression in tissue samples from patients with LN, there were 32 (52%) patients with grade 0, 16 (25%) patients with grade 1, and 14 (23%) patients with grade 2. Similar to the B-cell group, urine protein (g/24 hour), blood urea nitrogen, and serum creatinine levels were all higher in the BAFF expression group (*P* < 0.05 for all), with no statistically significant difference between grade 1 and grade 2 (*P* > 0.05 for all).

BAFF protein was mainly expressed in infiltrating inflammatory cells of the renal interstitial tissue, with very few seen in glomerular tissue (Figure 2).

Enhanced BAFF protein was most expressed in class IV LN (50%) and rare in class V LN (13%) (*P* = 0.0330 and *P* = 0.0227, resp.). The BAFF expression group demonstrated higher activity and chronicity indices than the non-BAFF expression group (*P* < 0.05 for all), with no significant difference between grades 1 and 2 (*P* > 0.05 for all).

### 3.6. Relationship between B-Cell Infiltration and BAFF Expression in the Renal Interstitium

**Figure 5:**
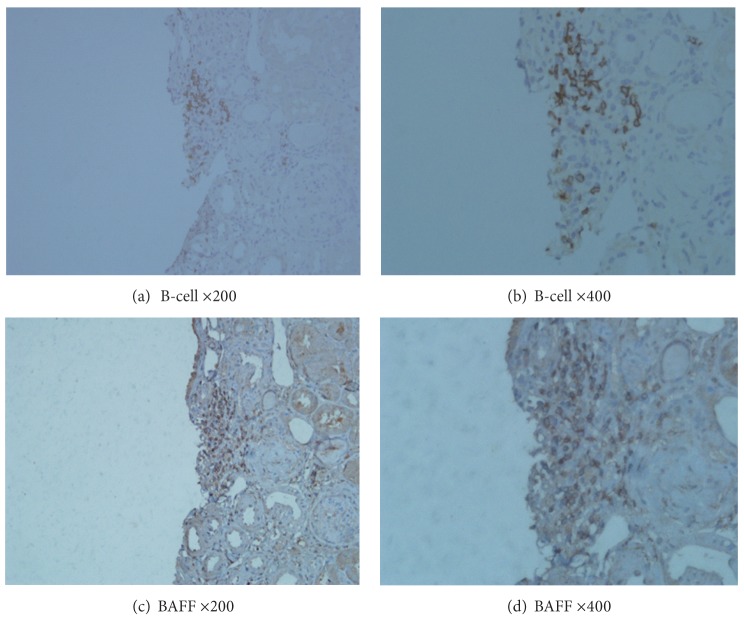
Local distribution of B cell and BAFF on renal biopsies with lupus nephritis. Immunohistochemistry was performed with monoclonal antibody against human CD20 (a), (b) and monoclonal antibody against BAFF (c), (d) on renal biopsies. These are consecutive samples from one patient. ((a)/(c) Original magnification: ×200; (b)/(d) original magnification: ×400).

The expression of BAFF was strongly associated with the amount of B-cell infiltration in the interstitium (*r* = 0.6226, *P* < 0.0001) (Table 3). There was evidence of strong expression of BAFF in the area of B-cell infiltrates in kidney samples from LN patients, although they did not completely overlap (Figure 5).

### 3.7. Relationship between Serum BAFF and Intrarenal BAFF Expression

There was no significant correlation between plasma BAFF levels and the degree of expression of BAFF protein in patients with LN (data not shown). 

### 3.8. Serum BAFF Levels in Different Groups

The levels of BAFF in the serum was determined in all 62 patients. There was no statistically significant difference between the B-cell infiltrating group and the non-B-cell infiltrating group (*P* > 0.05, data not shown). 

## 4. Discussion

Lupus nephritis is one of the most frequent and serious complications for patients with SLE and has a profound effect on both morbidity and mortality. Dysfunction of the B cells, an important component of adaptive immunity, is thought to be important in the pathogenesis of SLE/LN. The production of pathogenic antibody has been traditionally viewed as the principle contribution of B cells to the pathogenesis of immune-mediated glomerulonephritis [17]. However, it is increasingly appreciated that B cells have a much broader role in such diseases, functioning as antigen-presenting cells, regulators of T cells, macrophages, and dendritic cells, as well as being involved in the formation of local lymphatic expansion [3]. 

In this study, we prospectively analyzed 62 renal biopsies, which were then classified into different groups. As we have demonstrated, B cells are almost exclusively detectable in the tubulointerstitial compartment and rarely in the glomerular tuft. There was a significant correlation between intrarenal B cells and the degree of renal function. In our study, we found that proteinuria (g/24 hour), blood urea nitrogen, and serum creatinine levels were all significantly greater in the LN-B-cell group than in the LN-non-B-cell group. Furthermore, intrarenal B cells were more likely to be associated with class IV LN, and the activity and chronicity indices were also significantly higher in the LN-B-cell group compared with the LN-non-B-cell group. Therefore, it can be speculated that the formation of interstitial B-cell aggregates is a common response in LN and plays a pivotal role in renal injury. 

B cells are recruited to most chronically inflamed tissues, and resembling secondary lymphoid tissue has been found in several forms of inflammatory kidney diseases, including renal transplant rejection and lupus nephritis [18]. Their pathophysiological significance remains ill defined. Recent studies on LN patients have revealed that intrarenal B cells can form local lymphoid tissue, called tertiary lymphoid neogenesis, which display a mature non-antibody producing phenotype with antigen presenting ability [9]. Recently, Chang et al. reported that in some lupus renal biopsies there were germinal center- (GC-) like structures which were functional, as they were associated with in situ B-cell clonal expansion and somatic hypermutation [19]. In B-cell immunohistochemical staining, we found that 23 (37.1%) were of the scattered pattern (grade 1), and 12 (19.4%) demonstrated a nodular aggregates pattern (grade 2), which resembled germinal center- (GC-) like structures, suggesting ectopic germinal center formation, as mentioned previously. These different results may possibly reflect heterogeneity in LN pathogenesis and also emphasize the need for further research into the role of intrarenal B cells in LN.

As a new member of the TNF superfamily of ligands, B-cell activating factor (BAFF) was first discovered in 1999 and on binding to its receptors (BAFF-R) exerts strong stimulation on B cells, promoting B-cell survival and maturation [20]. APRIL (a proliferation-inducing ligand) considerable similarities with BAFF, APRIL is more important for plasma cells survival. But in the phenotype of APRIL transgenic (Tg) and APRIL knock-out mice is much less dramatic than that of the BAFF Tg and knock-out mice; thus we focus on BAFF. Increased serum and/or plasma levels of BAFF have been documented in several human autoimmune diseases such as SLE, rheumatoid arthritis, and Sjogren's syndrome, indicating the involvement of BAFF and the overactivation of B cells in the pathogenesis of these autoimmune diseases [21, 22]. Using immunohistochemical analysis, Groom et al. [23] demonstrated BAFF expression on infiltrating cells in salivary gland tissue from patients with Sjogren's syndrome, suggesting the importance of BAFF signaling in disease pathogenesis. In patients with rheumatoid arthritis, concentrations of BAFF in synovial membrane were much higher than in corresponding health control samples [22]. However, detailed data on local renal BAFF expression in human lupus nephritis is scarce. Neusser et al. [13] first demonstrated that BAFF and BAFF-R were expressed in interstitial inflammatory cell accumulates in LN patients. Zhao et al. [24] TACI expression tended to increase in SLE patients, especially in LN patients, which indicating that TACI may be a suppressive receptor of BAFF. Over-expression of TACI in patients with LN suggested that TACI may be involved in the pathogenesis of nephritis. In our study, we also found that BAFF was expressed in renal interstitial tissue, most infiltrated in class IV LN and has elevated proteinuria (g/24 h), blood urea nitrogen, and serum creatinine levels. Our experiment showed an abundant expression of BAFF in the area of B-cell infiltrates in renal interstitial tissue. The expression of BAFF was strongly associated with the amount of B-cell infiltration. Since the BAFF expression area did not exactly overlap with the B-cell infiltrates area, we cannot be certain that functional BAFF is released by activated B cells. In order to demonstrate that BAFF expression could be induced by infiltrating inflammatory cells, such as monocytes, macrophages, dendritic cells, and neutrophils, further research using confocal microscopy is needed. 

Clinical trials on anti-BAFF have been investigated in SLE, and the he blockade of the BAFF system appears to demonstrate some promising results [25]. Hence, BAFF-targeting therapy, by using therapeutic agents that block BAFF activity, is a promising area currently undergoing clinical trials, for the treatment of B-cell-related autoimmune diseases, particularly SLE.

Understanding the renal expression patterns of B cells and their effectors could help physicians in their choice of appropriate targeting reagents for different patients, but further research is needed in this area.

## 5. Conclusion

In conclusion, this study discussed correlations between clinical parameters, and the presence of different intrarenal B cell distributes in a large number of LN patients. As BAFF expression was strongly associated with B-cell infiltration, we hypothesize that altered B-cell differentiation and tolerance induced by excess BAFF may be central to the pathogenesis of LN pathogenesis. It would be interesting to study follow-up biopsies of these patients. Further research is needed to shed more light on the function of intrarenal B cells.

## Figures and Tables

**Table 1:** Demographic and clinical characteristics and laboratory findings of the LN patients.

	LN-B cell (*n* = 35)	LN-non-B cell (*n* = 27)	*P* value
Sex (male/female)	3/32	1/26	0.626
Age (years)	33 ± 11	29 ± 11	0.171
Prednisone dosage (mg/d)	30 ± 15	29 ± 14	0.514
Immunosuppressive drug*			
Cyclophosphamide	18	15	0.801
Azathioprine	6	4	1.000
Mycophenolate mofetil	9	8	0.779
SLE duration (months)	56 ± 54	49 ± 50	0.605
LN duration (months)	22 ± 36	19 ± 34	0.750
SLEDAI	13 ± 6	11 ± 5	0.221
Proteinuria (g/24 h)	3.93 ± 3.88	1.76 ± 1.34	0.010
Blood urea nitrogen (mmol/L)	9.41 ± 7.51	5.14 ± 1.81	0.006
Serum creatinine (*μ*mol/L)	80.60 ± 40.35	51.78 ± 10.35	0.001
Serum C3 (g/L)	0.49 ± 0.21	0.53 ± 0.20	0.443
Serum C4 (g/L)	0.095 ± 0.061	0.094 ± 0.060	0.958
Autoimmune antibody (positive/negative)			
Anti-dsDNA^†^	24/7	16/8	0.543
ANA^‡^	35/0	27/0	
Anti-Sm antibody	15/20	8/17	0.432
Anti-RNP antibody	16/19	14/13	0.798
Antinucleosome antibody	5/30	5/22	0.735

*One patient took cyclosporin A during the study.

^†^Anti-dsDNA antibodies were not detected in 4 patients in the LN-B-cell group and three patients in LN-non-B-cell group.

^‡^No statistical significance.

SLE: systemic lupus erythematosus; LN: lupus nephritis; SLEDAI: systemic lupus erythematosus disease activity index; anti-dsDNA: antidouble-stranded DNA antibody; ANA: antinuclear antibody; anti-RNP antibody: anti ribonucleoprotein antibody.

**Table 2:** Comparison between histological parameters of LN-B-cell and LN-non-B-cell groups.

	LN-B cell *n* = 35	LN-non-B cell *n* = 27	*P* value
ISN/RPS classification			<0.001*
I	0 (0.0)	3 (11.1)	
II	0 (0.0)	2 (7.4)	
III	3 (8.6)	1 (3.7)	
IV	17 (48.6)	5 (18.5)	
V	6 (17.1)	8 (29.6)	
III + V	4 (11.4)	7 (25.9)	
IV + V	5 (14.3)	1 (3.7)	
Activity index	6 (4–7)	4 (2–6)	0.033
Chronicity index	3 (1–5)	2 (0–2)	0.010

*P value for the difference in the ISN/RPS classification distribution between the two groups.

**Table 3:** Association between B-cell infiltration and BAFF expression.

	B-cell infiltration		
	Grade 0 (*n* = 27)	Grade 1 (*n* = 23)	Grade 2 (*n* = 12)
Intrarenal BAFF staining	—	—	—
No staining	24	7	1
Mild staining	2	9	5
Moderate to intense staining	1	7	6

B-cell infiltration was strongly associated with BAFF expression in the intrarenal region (*r* = 0.6226,  *P* < 0.0001).

## List of Abbreviations

SLE: Systemic lupus erythematosus
LN: Lupus nephritis
ANA: Antinuclear antibody
anti-dsDNA: Antidouble-stranded DNA antibody
SLEDAI: Systemic lupus erythematosus disease activity index
ISN/RPS: International Society of Nephrology/Renal Pathology Society
AI: Activity index
CI: Chronicity index
Scr: Serum creatinine
BUN: Blood urea nitrogen
ELISA: Enzyme-linked immunosorbent assay
PBS: Phosphate-buffered saline
RA: Rheumatoid arthritis
SS: Sjogren's syndrome
BLyS: B lymphocyte stimulator
BAFF: B-cell activating factor of the TNF family
BAFF-R: BAFF receptor
TACI: TNGR homology transmembrane activator and calcium modulator and cyclophilin ligand interactor
GC: Germinal center
FDC: Follicle dendritic cell
HRP: Horseradish peroxidase
TMB: Tetramethyl benzidine
HE: Hematoxylin and eosin
PAS: Periodic acid-Schiff
Masson: Masson's trichrome
PASM: Periodic acid-sliver methenamine.
