# Erratum to “The Characteristics and Significance of Locally Infiltrating B Cells in Lupus Nephritis and Their Association with Local BAFF Expression”

**DOI:** 10.1155/2016/7513892

**Published:** 2016-06-16

**Authors:** Chuan-Yin Sun, Yan Shen, Xiao-Wei Chen, Yu-Cheng Yan, Feng-Xia Wu, Ming Dai, Ting Li, Cheng-De Yang

**Affiliations:** ^1^Department of Rheumatology, Renji Hospital, Shanghai Jiaotong University School of Medicine, 145 Shan Dong Zhong Road, Shanghai 200001, China; ^2^Department of Rheumatology, First Affiliated Hospital of Medical School, Zhejiang University, 79 Qingchun Road, Hangzhou 310003, China; ^3^Department of Nephrology, Renji Hospital, Shanghai Jiaotong University School of Medicine, 145 Shan Dong Zhong Road, Shanghai 200001, China

The article titled “The Characteristics and Significance of Locally Infiltrating B Cells in Lupus Nephritis and Their Association with Local BAFF Expression” [1] listed the corresponding author incorrectly. The corresponding author of this paper is supposed to be Cheng-De Yang (yangchengde@hotmail.com) rather than Yu-ChengYan. The corrected author information is presented above.
